# Adaptation to the Information Society, Subjective Health Status, and Life Satisfaction Among Community-Dwelling Older Adults: A Cross-Sectional Secondary Analysis

**DOI:** 10.3390/healthcare14213825

**Published:** 2026-11-09

**Authors:** Seoyoung Park, Se-Won Kang, Ji-Ah Yun

**Affiliations:** 1 Department of Nursing, Gyeongsang National University, 861-15 Jinju-daero, Jinju-si 52727, Republic of Korea; masternur@gnu.ac.kr; 2 Department of Nursing, Dongseo University, 47 Jurye-ro, Busan 47011, Republic of Korea; wh3817@gdsu.dongseo.ac.kr

**Keywords:** information, health, life satisfaction, community, older adults

## Abstract

**Background/Objectives:** Community-dwelling older adults face challenges adapting to a rapidly changing information society. This study examined the associations of subjective health status and life satisfaction with adaptation to the information society. **Methods:** This cross-sectional secondary data analysis used data from the 2023 Survey of the Status of the Elderly in Korea (4 September–12 November 2023). The study included 10,078 adults aged ≥ 65 years. Statistical analyses, including the chi-square test, independent samples *t*-test, Pearson’s correlation coefficient, multiple regression analysis, and multinomial logistic regression analysis, were performed using IBM SPSS for Windows. **Results:** Adaptation to the information society differed significantly according to general and information-related characteristics (all *p* < 0.001). Adaptation was positively correlated with subjective health status (*r* = 0.362, *p* < 0.001) and life satisfaction (*r* = 0.242, *p* < 0.001). Significant differences in subjective health status (*t* = −29.193, *p* < 0.001) and life satisfaction (*t* = −17.738, *p* < 0.001) were observed between adaptation groups. Multiple regression analysis indicated that subjective health status (β = 0.178, *p* < 0.001) and life satisfaction (β = 0.073, *p* < 0.001) were significantly associated with adaptation to the information society. The overall regression model explained 25.0% of the variance in adaptation to the information society (adjusted R^2^ = 0.250, F = 332.284, *p* < 0.001). Multinomial logistic regression analysis showed that subjective health status (OR = 2.313, 95% CI = 2.008–2.665, *p* < 0.001) and life satisfaction (OR = 1.449, 95% CI = 1.187–1.767, *p* < 0.001) were significantly associated with higher odds of belonging to the good adaptation group. **Conclusions:** Subjective health status and life satisfaction were significantly associated with adaptation to the information society among community-dwelling older adults. These findings suggest the importance of digital education, health promotion, and well-being support.

## 1. Introduction

The global population is aging rapidly owing to advancements in medical technology and increased life expectancy [1]. In particular, Korea is aging at an exceptionally rapid rate, with the proportion of the population aged 65 and older increasing from 15.73% in 2020 to 20.3% in 2025, entering a super-aged society [2]. Furthermore, Korea’s rate of population aging exceeds the average among OECD member countries, and Korea is expected to become one of the most aged societies among OECD nations in the future [3]. This rapid aging not only reflects a change in population structure but also highlights the need for new societal responses in areas such as social welfare, quality of life, and health management for older adults.

Modern society is rapidly transitioning into an information society centered on digital technology, including mobile healthcare, non-face-to-face counseling, remote monitoring, and health-management applications. In an information society, the ability to utilize digital technology, including digital devices and online services, across all aspects of daily life is considered important. Consequently, an individual’s level of adaptation to an information society has a significant impact on social participation and health management [4,5]. Adaptation to an information society refers to the ability to access, understand, and utilize information in a digital environment and respond appropriately in daily life and social relationships. This concept is related to digital literacy and digital competency. The higher the level of adaptation to the information society, the more proficient individuals are in utilizing digital technology, thereby increasing the likelihood of social participation and effective health management [6].

However, the use of digital technology is influenced by the digital divide, which varies according to age, educational level, economic status, and confidence in using technology [7,8]. In particular, older adults experience greater difficulty adapting to the digital environment than other age groups because of physical decline, reduced sensory and cognitive functions, and limited experience using digital devices [9,10]. The digital divide can exacerbate social isolation and inequality among older adults by limiting access to information, reducing social participation, and creating difficulties in accessing welfare services [9].

Digital literacy among older adults has been shown to positively affect access to health information, self-health management, maintenance of social support networks, and reduced depression and social isolation. Higher digital literacy among older adults was associated with better self-rated health status, lower levels of depression, and greater life satisfaction [11,12]. In other words, older adults with a high adaptation to the information society are more likely to use digital technology proficiently. These findings suggest that adapting to an information society is associated with subjective health and life satisfaction.

Previous studies have examined various factors related to older adults’ adaptation to the information society and digital literacy. These studies have primarily focused on the relationships between sociodemographic characteristics such as gender, age, educational level, economic status, and experience using digital devices. Domestic studies have indicated that the digital divide is smaller among men than among women, and younger individuals have higher educational levels and report better subjective health status [13]. Low educational levels among older adults have also been reported as a major factor exacerbating digital inequality [14]. International studies have shown that digital literacy is lower among individuals with lower educational levels (below the middle school level), those requiring assistance with activities of daily living, and those who participate less in social activities [12]. Age, income, educational level, occupation, and interest in technology were identified as the major predictors of digital literacy among older adults [15]. Furthermore, high levels of digital device use among older adults have been associated with successful aging and high life satisfaction [16], and social interaction through digital device use reduces depression [17]. However, research on information society adaptation among community-dwelling older adults remains limited and requires a multifaceted approach. In particular, studies should focus on variables that explain the adaptation to the information society in terms of the social integration of older adults.

This study focused on subjective health status and life satisfaction. Examining the digital adaptation of older adults solely in terms of their technical skills is insufficient. To explain their social integration, it is necessary to consider both their subjective health status, which may influence their capacity for adaptation, and life satisfaction, as potential outcomes of such adaptation. Consequently, the findings may inform policies that extend beyond simple educational programs to include health promotion, social support, and improved quality of life.

While adaptation to the information society is conceptually related to digital literacy and digital competence, these concepts are not identical. Digital literacy generally refers to the ability to access, understand, evaluate, and utilize digital information, whereas digital competence encompasses a broader range of knowledge, skills, and attitudes required for effective engagement in digital environments. In contrast, adaptation to the information society reflects the extent to which individuals successfully apply these abilities in their daily lives and participate in social activities within a rapidly evolving digital society. Although previous studies have examined digital literacy and its associations with health and well-being among older adults, research focusing on adaptation to the information society remains limited. Furthermore, few studies have simultaneously examined subjective health status and life satisfaction in relation to adaptation to the information society using a large nationally representative sample of community-dwelling older adults. Therefore, this study contributes to the existing literature by addressing this gap using nationally representative data from older adults in Korea.

### Study Purpose

This study aimed to identify the relationships among information society adaptation, subjective health status, and life satisfaction among community-dwelling older adults. The specific objectives of this study are as follows:

1. To identify differences in information society adaptation according to participants’ characteristics.
2. To identify the relationships among participants’ information society adaptation, subjective health status, and life satisfaction.
3. To identify differences in subjective health status and life satisfaction according to participants’ level of information society adaptation.
4. To identify subjective health status and life satisfaction as factors associated with participants’ information society adaptation.

## 2. Materials and Methods

### 2.1. Study Design

This secondary data analysis study aimed to identify the relationships between information society adaptation, subjective health status, and life satisfaction among community-dwelling older adults.

### 2.2. Study Participants

We analyzed data from all 10,078 participants aged ≥ 65 years who participated in the 2023 Survey of the Status of the Elderly in Korea. No additional inclusion or exclusion criteria were applied. Cases with missing data on adaptation to the information society, subjective health status, or life satisfaction were excluded from the relevant analyses, resulting in slight variations in sample size across analyses and tables.

### 2.3. Data Collection

The source data for this study were obtained from the 2023 Survey on the Status of the Elderly and were utilized in accordance with the data usage procedures of the Health and Welfare Data Portal of the Korea Institute for Health and Social Affairs (KIHASA) [18]. This statutory survey, conducted every three years under Article 5 of the Elderly Welfare Act since 2007, investigates the living conditions and welfare needs of older adults in Korea and provides baseline data for policy development and academic research on population aging.

Raw data were collected using stratified cluster sampling. The first stratification level was based on cities and provinces, the second on dongs and eups/myeons, and the third on survey area characteristics. Specifically, the first level included 17 cities and provinces, the second level included dongs and eups/myeons, and the third level included general and apartment survey areas as stratification variables. In the second level of stratification, the eup/myeon areas in special cities, metropolitan cities, and Sejong City were integrated and classified as dongs, whereas the nine provincial regions were stratified as dongs and eups/myeons, resulting in 52 strata. Data were collected by 176 trained interviewers using tablet PC-assisted personal interviews (TPP) through one-on-one, face-to-face interviews conducted between 4 September and 12 November 2023.

### 2.4. Study Variables

#### 2.4.1. Information Society Adaptation

Information society adaptation was measured using a single item assessing the degree of adaptation perceived by older adults: “Do you find it difficult to adapt to the rapidly developing information society?” Responses were rated on a 5-point Likert scale, with 5 indicating “not difficult at all,” 4 “not difficult,” 3 “average,” 2 “difficult,” and 1 “very difficult.” Higher scores indicate better adaptation to an information society.

For group comparisons, participants with scores of 3 or higher were classified as the good group, whereas those with scores below 3 were classified as the risk group, based on the midpoint of the 5-point Likert scale, with a score of 3 representing an average level of adaptation.

#### 2.4.2. Subjective Health Status

Subjective health status refers to an individual’s perception of their own health. It was measured using a single item: “How do you think your usual health status is?” Responses were rated on a 5-point Likert scale, with 5 indicating “very healthy,” 4 “fairly healthy,” 3 “average,” 2 “fairly unhealthy,” and 1 “not healthy at all.” Higher scores indicate better subjective health status.

#### 2.4.3. Life Satisfaction

Life satisfaction was measured using a single item assessing overall satisfaction with life: “How satisfied are you with your current life overall?” Responses were rated on a 5-point Likert scale, with 5 indicating “very satisfied,” 4 “satisfied,” 3 “average,” 2 “dissatisfied,” and 1 “very dissatisfied.” Higher scores indicate greater life satisfaction.

#### 2.4.4. General Characteristics

General characteristics included sex, age, education level, employment status, cohabitation type, annual household income, and functional status. Age was classified as early (65–74 years), middle (75–84 years), or late (≥85 years) old age [19,20]. Educational level was categorized as elementary school or lower, middle school, or high school or higher. Employment status was classified as employed or unemployed, based on engagement in income-generating work. The cohabitation type was classified as living alone, living with a spouse only, or living with family members or others. Annual household income was categorized as <50%, 50% to <100%, 100% to <150%, or ≥150% of the median household income. Functional status was measured using activities of daily living (ADLs) and instrumental activities of daily living (IADLs) [21,22]. The ADL and IADL instruments were scored as “complete independence,” “partial assistance,” or “complete assistance” based on responses to the question, “How much assistance did you need from others to perform each item during the past week?” The ADL scale consisted of seven items and the IADL scale consisted of 10 items. Participants were classified as independent if all items were rated as “complete independence,” and as dependent if they required “partial assistance” or “complete assistance” for at least one item. In this study, participants who were not completely independent in at least one item were classified as having a functional limitation. The seven ADL items included dressing, washing the face, brushing teeth or hair, bathing or showering, eating prepared food, lying down and getting up to leave the room, using the toilet, managing post-urination or post-defecation care, and bowel and bladder control. The 10 IADL items included grooming, housework, meal preparation, laundry, shopping, money transactions, making and receiving telephone calls, taking medications, financial management, short-distance outings, and transportation.

#### 2.4.5. Information-Related Characteristics

Information-related characteristics included ownership of digital devices, smartphone use, computer use, smartwatch use, household Internet access, and scope of digital device use. The survey assessed activities, such as receiving and sending messages, video calls, information search and retrieval, taking photographs or videos, listening to music, playing games, watching videos, using social media, e-commerce, financial transactions, searching for and installing applications, and placing and receiving orders using kiosks.

### 2.5. Ethical Considerations

The original data collection used in this study was processed with the approval of the Institutional Review Board (IRB) of the KIHASA (Notification of Review Results No. 2023-078). The study was conducted by trained researchers, who obtained written informed consent from all participants prior to data collection. In this secondary analysis, ethical review and approval were waived because the study utilized previously collected data and publicly available datasets. Raw data are publicly available through the KIHASA Health and Welfare Data Portal (https://www.kihasa.re.kr/dataportal, accessed on 30 September 2026) after a simple approval process. However, participant identification was not possible because the dataset did not contain personally identifiable information. Permission to use the data appropriate for the study objectives and participant selection criteria was obtained through the required approval process.

### 2.6. Data Analysis

Data were analyzed using IBM SPSS Statistics (version 23.0; IBM, Armonk, NY, USA).

1. Frequencies, percentages, means, and standard deviations were used to analyze participants’ characteristics.
2. Differences in information society adaptation according to participant characteristics were analyzed using an independent samples *t*-test and a one-way analysis of variance (ANOVA). The relationships among information society adaptation, subjective health status, and life satisfaction were analyzed using Pearson’s correlation coefficients.
3. Differences in subjective health status and life satisfaction according to the information society adaptation groups were analyzed using frequencies, percentages, means, standard deviations, chi-square tests, and independent sample *t*-tests.
4. Multiple and multinomial logistic regression analyses were performed to examine the relationships among information society adaptation, subjective health status, and life satisfaction.
5. All analyses were conducted using the sampling weights provided in the original survey data. The original survey employed a stratified cluster sampling design.
6. Statistical significance was set at *p* < 0.05.

## 3. Results

### 3.1. Differences in Information Society Adaptation According to Participants’ Characteristics

Participants’ characteristics and the derived differences in information society are presented in Table 1. Among the 10,078 participants, 3872 (38.4%) were men, the mean age was 74.13 years, and 5731 (56.9%) were aged 65–74 years. Regarding educational level, 4449 participants (44.1%) had completed elementary school or lower, and regarding employment status, 3948 (39.2%) were employed. Regarding cohabitation, 5480 (54.4%) lived with their spouses only. Regarding annual household income, 4015 (39.8%) had an income of less than 50% of the median household income, and regarding functional status, 1762 (17.5%) had functional limitations.

Regarding information-related characteristics, 6432 participants (63.8%) used smartphones, 1099 (10.9%) used computers, 146 (1.4%) used smartwatches, and 4295 (42.6%) had internet access at home. Regarding the scope of digital device usage, 8120 participants (80.6%) reported receiving messages, 7021 (70.6%) sending messages, 4158 (41.8%) making video calls, 4626 (46.5%) searching for information, 4895 (49.2%) taking photos or videos, 2436 (24.5%) listening to music, 1325 (13.3%) playing games, 4077 (41.0%) watching videos, 801 (8.0%) using social media, 1074 (10.8%) engaging in e-commerce, 1782 (17.9%) conducting financial transactions, 1181 (11.9%) searching for and installing applications, and 1529 (15.4%) placing and receiving orders using kiosks.

Significant differences in information society adaptation according to participants’ characteristics were found for all general characteristics, including sex, age, educational level, employment status, cohabitation type, annual household income, and functional status (all *p* < 0.001). Significant differences were also found for all information-related characteristics, including smartphone, computer, and smartwatch use, household Internet access, and the scope of digital device use (all *p* < 0.001) (Table 1).

### 3.2. Relationship Among Participants’ Information Society Adaptation, Subjective Health Status, and Life Satisfaction

The relationships between information society adaptation, subjective health status, and life satisfaction are presented in (Table 2). Information society adaptation was positively correlated with subjective health status (*r* = 0.362, *p* < 0.001) and life satisfaction (*r* = 0.242, *p* < 0.001). Subjective health status was also positively correlated with life satisfaction (*r* = 0.419, *p* < 0.001).

The mean ± standard deviation score for information society adaptation was 2.09 ± 0.91 (out of 5), whereas the mean scores for subjective health status and life satisfaction were 3.17 ± 0.89 and 3.32 ± 0.66 (out of 5), respectively.

### 3.3. Differences in Subjective Health Status and Life Satisfaction According to Participants’ Information Society Adaptation Groups

To examine differences in subjective health status and life satisfaction according to the information society adaptation group, participants with scores of 3 or higher on the 5-point scale were classified as the “good group,” whereas those with scores below 3 were classified as the “risk group.” The mean scores and distributions were compared between the groups (Table 3).

Regarding subjective health status, there was a difference in the mean scores between the good and risk groups (3.55 > 3.00, t = −29.193, *p* < 0.001). A significant difference in the distribution of subjective health status was also observed between the groups (χ^2^ = 490.914, *p* < 0.001).

Regarding life satisfaction, there was a difference in the mean scores between the good and risk groups (3.50 > 3.25, t = −17.738, *p* < 0.001). The distribution of life satisfaction also differed significantly between the groups (χ^2^ = 83.153, *p* < 0.001).

### 3.4. Subjective Health Status and Life Satisfaction as Factors Associated with Participants’ Information Society Adaptation

Multiple and multinomial logistic regression analyses were performed to examine the relationships among information society adaptation, subjective health status, and life satisfaction. The analyses were conducted after controlling for general characteristics (sex, age, educational level, employment status, cohabitation type, annual household income, and functional status), which showed differences in information society adaptation in basic statistics.

The results of the multiple regression analysis are presented in Table 4. Subjective health status (β = 0.178, *p* < 0.001) and life satisfaction (β = 0.073, *p* < 0.001) were significantly associated with information society adaptation. The overall regression model explained 25.0% of the variance in information society adaptation (adjusted R^2^ = 0.250, F = 332.284, *p* < 0.001). The regression model was stable and showed no evidence of multicollinearity (tolerance = 0.61–0.85, variance inflation factor = 1.17–1.62).

In addition, multinomial logistic regression analysis was conducted to examine the associations of subjective health status and life satisfaction with information society adaptation, and the results are presented in Table 5. Participants with good subjective health status had 2.313 times higher odds of belonging to the good adaptation group than those with poor subjective health status (95% CI, 2.008–2.665; *p* < 0.001). Similarly, participants with good life satisfaction had 1.449 times higher odds of belonging to the good adaptation group than those with poor life satisfaction (95% CI, 1.187–1.767; *p* < 0.001).

## 4. Discussion

This study was conducted to identify the relationships among information society adaptation, subjective health status, and life satisfaction among community-dwelling older adults and to examine whether subjective health status and life satisfaction are associated with information society adaptation. The results showed significant differences in the degree of information society adaptation according to the general characteristics of community-dwelling older adults, and identified subjective health status and life satisfaction as significant factors associated with information society adaptation.

### 4.1. Differences in Information Society Adaptation According to Participants’ Characteristics

This study found significant differences in the degree of information society adaptation according to general and information-related characteristics of community-dwelling older adults. Regarding general characteristics, men, younger age groups (65–74 years), those with high school education or higher, employed individuals, those living with a spouse, those with a household income above 150% of the median income, and those without functional limitations had significantly higher information society adaptation scores than their counterparts. Similarly, regarding information-related characteristics, those who used digital devices (e.g., smartphones, computers, smartwatches, and household Internet services) demonstrated significantly higher adaptation scores than non-users.

These findings are consistent with those of Jang [13], who reported that the digital divide is smaller among men, younger age groups, and individuals with higher educational attainment, better health, and higher economic status. These findings are also consistent with reports that digital literacy is lower among older adults with lower educational attainment, those requiring assistance with daily living, and those who participate less in social activities [12]. Similarly, age, income, educational level, and occupation have been identified as major predictors of digital literacy among older adults [11]. These differences may be attributable to unequal access to digital resources and opportunities for technology use. Older adults with higher educational attainment and income may have greater exposure to digital technologies and more opportunities to develop digital competencies. Similarly, functional limitations may create barriers to technology use and reduce confidence in navigating digital environments. In other words, because differences in the sociodemographic characteristics of older adults can contribute to the digital divide, policies are needed to provide tailored digital education to groups vulnerable to the digital divide, such as those with low income, low educational attainment, and unemployment status.

### 4.2. Relationship Among Information Society Adaptation, Subjective Health Status, and Life Satisfaction

This study identified significant positive correlations between information society adaptation, subjective health status, and life satisfaction, with a particularly strong positive correlation between subjective health status and life satisfaction. These findings indicate that older adults who perceive themselves as healthier and are more satisfied with their lives are better able to adapt to information and digital environments.

These findings are consistent with those of Lee et al. [23], who reported that higher digital literacy promotes healthy lifestyle behaviors, and Jung et al. [24], who found that digital literacy positively influences life satisfaction. Taken together, these findings suggest that adaptation to an information society among older adults extends beyond digital technology use and is associated with health perceptions and quality of life.

Notably, the mean score for information society adaptation was relatively low (2.09 out of 5), suggesting that many older adults continue to experience difficulties adapting to a rapidly evolving digital environment. Given the increasing reliance on digital technologies in healthcare, welfare services, and social communication, this finding highlights the need for continued efforts to reduce digital exclusion and support digital engagement among older adults.

### 4.3. Differences in Subjective Health Status and Life Satisfaction According to Participants’ Information Society Adaptation Group

In this study, the “good” information society adaptation group demonstrated significantly higher subjective health status and life satisfaction than the “risk” group. Furthermore, significant differences in the distribution of subjective health status and life satisfaction were observed between the two groups. These findings are consistent with those of a literature review that identified health-related barriers as major obstacles to digital literacy among older adults [9]. Similarly, Gordon and Hornbrook [25] reported that a higher prevalence of chronic diseases was associated with greater difficulty in using digital health information, whereas Jung et al. [26] found that digital literacy affects life satisfaction. Therefore, differences in the level of societal adaptation to information may reflect differences in the health and psychosocial well-being of older adults.

### 4.4. Subjective Health Status and Life Satisfaction as Factors Related to Participants’ Information Society Adaptation

In this study, multiple regression analysis controlling for general characteristics showed that subjective health status and life satisfaction were significantly associated with information society adaptation, with subjective health status showing a strong association. The stronger association between subjective health status and information society adaptation compared with life satisfaction may be explained by the functional and cognitive resources required for digital engagement. Older adults who perceive themselves as healthier may have greater physical capacity, cognitive functioning, and confidence to learn and use digital technologies. In contrast, life satisfaction reflects a broader evaluation of personal well-being and may therefore have a more indirect relationship with adaptation to the information society. Furthermore, multinomial logistic regression analysis demonstrated that participants with good subjective health status had 2.313 times higher odds of belonging to the good information society adaptation group than those with poor subjective health status. Similarly, participants with good life satisfaction had 1.449 times higher odds of belonging to the good adaptation group than those with poor life satisfaction.

These findings suggest that adaptation to the information society among older adults is associated not only with digital literacy but also with perceived health and psychological well-being. Recent international studies have reported that digital literacy should be explained not only by structural variables such as education and income but also by social participation, functional status, and psychological resources [7]. Khan et al. [9] and Kim [27] further suggested that improving digital literacy among older adults requires consideration of not only technical education but also motivation for technology use and broader life contexts. Accordingly, the present findings support the need for an integrated approach that includes health promotion and emotional well-being enhancement to facilitate adaptation to the information society among older adults.

This study has several strengths. First, it extends previous research by considering subjective health status and life satisfaction in addition to sociodemographic characteristics when explaining information society adaptation among community-dwelling older adults. This broadens the concept of information society adaptation beyond digital literacy to include health management and psychosocial wellbeing. Second, this study used nationally representative data from the 2023 Survey on the Status of the Elderly. By demonstrating that subjective health status and life satisfaction were significantly associated with information society adaptation, this study highlights the importance of health and psychosocial factors in explaining digital adaptation among older adults. Third, this study provides foundational evidence for the development of community-based health promotion policies and strategies to reduce the digital divide by demonstrating that differences in information society adaptation are associated with differences in health and quality of life.

### 4.5. Limitations of the Study

This study has the advantage of a high sample representativeness because it used data from the Survey on the Status of the Elderly; however, it has several limitations. First, because this was a cross-sectional study that used secondary data, causal relationships among the variables could not be established. Subjective health status is considered an ‘internal resource’ for information society adaptation, whereas life satisfaction is considered an ‘external outcome’ related to older adults’social integration. However, the direction of these relationships cannot be determined based on the current findings alone. Therefore, causal relationships among subjective health status, life satisfaction, and information society adaptation cannot be inferred from the present findings. Future studies should use path analysis to verify the causal relationships among these variables. Second, information society adaptation was measured using a single self-reported item, which may not adequately reflect the multidimensional nature of digital literacy or actual digital skills. Therefore, the measure may represent perceived adaptation rather than objectively assessed digital competence. Third, the classification of information society adaptation, subjective health status, and life satisfaction into categorical groups for some analyses may have reduced variability in the original data and resulted in a loss of information. Fourth, as this study included only community-dwelling older adults, the findings may not be generalizable to older adults living in long-term care facilities or those with severe functional limitations.

## 5. Conclusions

This study identified the relationships among information society adaptation, subjective health status, and life satisfaction among community-dwelling older adults and examined the associations of subjective health status and life satisfaction with information society adaptation. Information society adaptation differed according to participants’ general and information-related characteristics and was positively correlated with subjective health status and life satisfaction. Subjective health status and life satisfaction were significantly associated with information society adaptation, with subjective health status showing the strongest association. These findings suggest that health promotion and emotional well-being, along with digital education, may be important considerations when developing strategies to support adaptation to the information society among community-dwelling older adults.

Based on these findings, the following recommendations are proposed. First, programs aimed at supporting adaptation to the information society among community-dwelling older adults may benefit from incorporating health management and psychosocial support in addition to digital device education. Second, policymakers and practitioners may consider prioritizing older adults who are vulnerable to the digital divide, including those with advanced age, low educational attainment, low income, and functional limitations. Third, future studies should use multidimensional measures of information society adaptation and digital competence and employ longitudinal research designs to better understand the relationships among these variables. Finally, replication studies involving diverse populations, including older adults residing in long-term care facilities and those with severe functional limitations, are needed to improve the generalizability of the findings.

## Figures and Tables

**Table 1:** Information society adaptation according to participants’ characteristics.

Variables			N = 10,078, *n* (%)	Information Society Adaptation	
				Mean (SD)	t(*p*)/F(*p*)
**General characteristics**					
Sex		Male	3872 (38.4)	2.25 (0.91)	13.965 (<0.001)
		Female	6206 (61.6)	1.99 (0.89)	
Age (years)		65–74 ^a^	5731 (56.9)	2.36 (0.90)	667.817 (<0.001) a > b > c
		75–84 ^b^	3494 (34.7)	1.79 (0.80)	
		≥85 ^c^	853 (8.5)	1.53 (0.73)	
		Mean ± SD (range, min–max)	74.13 ± 6.81 (65–103)		
Educational level		≤Elementary school ^a^	4449 (44.1)	1.73 (0.80)	865.926 (<0.001) a < b < c
		Middle school ^b^	2127 (21.1)	2.13 (0.84)	
		High school ^c^	3502 (34.7)	2.52 (0.88)	
Employment status		Employed	3948 (39.2)	2.22 (0.93)	11.390 (<0.001)
		Unemployed	6130 (60.8)	2.01 (0.89)	
Cohabitation type		Living alone ^a^	3448 (34.2)	1.92 (0.88)	109.431 (<0.001) b > c > a
		Living with a spouse only ^b^	5480 (54.4)	2.21 (0.91)	
		Living with family members or others ^c^	1150 (11.4)	2.04 (0.90)	
Annual household income * (based on median income)		<50% ^a^	4015 (39.8)	1.87 (0.84)	244.233 (<0.001) a < b< c < d
		50% to <100% ^b^	2910 (28.9)	2.05 (0.89)	
		100% to <150% ^c^	1569 (15.6)	2.32 (0.90)	
		≥150% ^d^	1584 (15.7)	2.51 (0.91)	
Functional status		Functional limitation	1762 (17.5)	1.65 (0.78)	−22.454 (<0.001)
		No functional limitation	8316 (82.5)	2.18 (0.91)	
**Information-related characteristics**					
Smartphone use		Yes	6432 (63.8)	2.35 (0.89)	40.929 (<0.001)
		No	3646 (36.2)	1.63 (0.76)	
Computer use		Yes	1099 (10.9)	2.78 (0.88)	27.621 (<0.001)
		No	8979 (89.1)	2.01 (0.88)	
Smartwatch use		Yes	146 (1.4)	3.04 (0.85)	13.502 (<0.001)
		No	9931 (98.6)	2.08 (0.90)	
Household Internet access		Yes	4295 (42.6)	2.40 (0.89)	30.578 (<0.001)
		No	5783 (57.4)	1.86 (0.85)	
Digital device use *	① Receiving messages	Yes	8120 (80.6)	2.24 (0.89)	36.421 (<0.001)
		No	1831 (18.4)	1.44 (0.68)	
	② Sending messages	Yes	7021 (70.6)	2.34 (0.87)	47.390 (<0.001)
		No	2930 (29.4)	1.49 (0.70)	
	③ Video calls	Yes	4158 (41.8)	2.45 (0.88)	35.699 (<0.001)
		No	5793 (58.2)	1.83 (0.84)	
	④ Information search and retrieval	Yes	4626 (46.5)	2.52 (0.84)	48.488 (<0.001)
		No	5325 (53.5)	1.72 (0.80)	
	⑤ Taking photographs and videos	Yes	4895 (49.2)	2.42 (0.87)	38.243 (<0.001)
		No	5056 (50.8)	1.77 (0.83)	
	⑥ Listening to music	Yes	2436 (24.5)	2.60 (0.89)	33.376 (<0.001)
		No	7517 (75.5)	1.93 (0.85)	
	⑦ Gaming	Yes	1325 (13.3)	2.79 (0.83)	32.671 (<0.001)
		No	8626 (86.7)	1.99 (0.87)	
	⑧ Watching videos	Yes	4077 (41.0)	2.51 (0.88)	41.682 (<0.001)
		No	5874 (59.0)	1.80 (0.81)	
	⑨ Social media use	Yes	801 (8.0)	2.92 (0.86)	28.212 (<0.001)
		No	9150 (92.0)	2.02 (0.88)	
	⑩ E-commerce	Yes	1074 (10.8)	2.94 (0.84)	34.837 (<0.001)
		No	8877 (89.2)	1.99 (0.86)	
	⑪ Financial transactions	Yes	1782 (17.9)	2.77 (0.85)	37.272 (<0.001)
		No	8169 (82.1)	1.94 (0.85)	
	⑫ Application search and installation	Yes	1181 (11.9)	2.87 (0.85)	33.458 (<0.001)
		No	8769 (88.1)	1.99 (0.86)	
	⑬ Kiosk use (placing and receiving orders)	Yes	1529 (15.4)	2.74 (0.86)	31.743 (<0.001)
		No	8422 (83.6)	1.98 (0.87)	

SD, standard deviation; * Estimated average median income: <50%, 10.446 million KRW; 50% to <100%, 27.194 million KRW; 100% to <150%, 46.272 million KRW; ≥150%, 85.631 million KRW; cf. Estimated mean annual household income: 34.686 million KRW. * excluding missing values.

**Table 2:** Correlations among variables.

Variables (Range: 1–5)	Information Society Adaptation	Subjective Health Status	Life Satisfaction
	*r*(*p*)		
Information society adaptation Mean (SD), 2.09 (0.91)	1		
Subjective health status Mean (SD), 3.17 (0.89)	0.362 (<0.001)	1	
Life satisfaction Mean (SD), 3.32 (0.66)	0.242 (<0.001)	0.419 (<0.001)	1

SD. Stadard Deviation.

**Table 3:** Subjective health status and life satisfaction according to participants’ information society adaptation groups.

Variables		Information Society Adaptation			χ2 or t(*p*)
		Total ^a^ (*n* = 9951)	Good Group ^b^ (*n* = 3020, 30.3%)	Risk Group ^C^ (*n* = 6931, 69.7%)	
		*n* (%) or Mean(SD)			
Subjective health status	Good group ^b^	7558 (76.0)	2728 (36.1)	4830 (63.9)	490.814 (<0.001)
	Risk group ^C^	2393 (24.0)	292 (12.2)	2101 (87.8)	
	Mean (SD)	3.17 (0.89)	3.55 (0.76)	3.00 (0.89)	−29.193 (<0.001)
Life satisfaction	Good group ^b^	9093 (91.4)	2877 (31.6)	6216 (68.4)	83.153 (<0.001)
	Risk group ^C^	858 (8.6)	143 (16.7)	715 (83.3)	
	Mean (SD)	3.32 (0.66)	3.50 (0.64)	3.25 (0.66)	−17.738 (<0.001)

^a^ excluding missing values; ^b^ Good group: ≥3 points; ^c^ Risk group, <3 points (range: 1–5).

**Table 4:** Multiple Regression Analysis of Information Society Adaptation According to Subjective Health Status and Life Satisfaction.

Variables	Information Society Adaptation				
	B	SE	β	t	*p*
Subjective health status	0.182	0.011	0.178	17.081	<0.001
Life satisfaction	0.100	0.013	0.073	7.553	<0.001

R^2^ = 0.251; Adjusted R^2^ = 0.250; F = 332.284 (*p* < 0.001); Tolerance = 0.61–0.85; Variance inflation factor = 1.17–1.62, SE, standard error; Adjusted variables included sex, age, educational level, employment status, current job, cohabitation type, annual household income, and functional status.

**Table 5:** Multinomial logistic regression analysis of information society adaptation according to subjective health status and life satisfaction.

Variables		Information Society Adaptation ^a^			
		B	*p*	OR	95% CI
Subjective health status	Good group ^b^	0.839	<0.001	2.313	2.008–2.665
	Risk group ^c^			1	
Life satisfaction	Good group ^b^	0.371	<0.001	1.449	1.187–1.767
	Risk group ^c^			1	

OR, odds ratio; CI, Confidence Interval; ^a^ compare group-good group, baseline group-risk group; ^b^ Good group: ≥3 points; ^c^ Risk group, <3 points (range: 1–5); Adjusted variables included sex, age, educational level, employment status, cohabitation type, current job, living together, annual household income, and functional status.

## Data Availability

The data presented in this study are available from KIHASA Health and Welfare Data Portal (https://www.kihasa.re.kr/dataportal).
